# Effect of Thermal Post-Treatment on the Mechanical Performance and Microstructure of Modified Photosensitive PLA/Starch Blends Obtained by Digital Light Processing

**DOI:** 10.3390/polym18070836

**Published:** 2026-03-29

**Authors:** Mustapha Nouri, Sofiane Belhabib, Mahfoud Tahlaiti, Jaianth Vijayakumar, Elodie Boller, Sofiane Guessasma

**Affiliations:** 1 Icam, Site de Nantes, 35 Av. du Champ de Manoeuvres, 44470 Carquefou, France; mustapha.nouri@icam.fr (M.N.); sofiane.belhabib@univ-nantes.fr (M.T.); 2 GeM, Ecole Centrale Nantes, CNRS, UMR 6183, Nantes Université, 44000 Nantes, France; 3 Nantes Université, IUT, 2 Av. du Professeur Jean Rouxel, 44470 Carquefou, France; mahfoud.tahlaiti@icam.fr; 4 The European Synchrotron (ESRF), 71 Avenue des Martyrs, 38000 Grenoble, France; jaianth.vijayakumar@esrf.fr (J.V.); boller@esrf.fr (E.B.); 5 INRAE, Research Unit BIA UR1268, Rue Geraudiere, 44316 Nantes, France

**Keywords:** digital light processing, photosensitive polylactic acid, starch, tensile performance, synchrotron X-ray-microtomography

## Abstract

We investigate 3D-printed composite materials composed of a photosensitive polylactic acid (PLA) resin blended with 10% starch and fabricated by Digital Light Processing. We synthesize the 3D-printed composites by incorporating a post-processing stage involving thermomoulding at various temperatures ranging from 50 °C to 150 °C. The composition, structure, and thermal and mechanical performance of the 3D-printed composites are evaluated using infrared spectroscopy (FTIR), Differential Scanning Calorimetry (DSC), synchrotron X-ray microtomography and tensile testing assisted with digital image correlation. Our results indicate that post-treatment influences the mechanical behaviour of the composites, leading to a moderate increase in stiffness while the tensile strength remains slightly reduced compared with the reference condition, particularly when moulding temperatures reach 100 °C. Our 3D printing approach combined with the photosensitive/starch blend provides a cost-effective alternative for obtaining 3D-printed biosourced components, maintaining technical performance at a reasonable cost.

## 1. Introduction

Additive manufacturing (AM) has emerged as a ground-breaking technology that has garnered significant attention in recent decades [1,2]. It holds great promise as a processing technology for designing intricately complex technical parts [3]. A widely accepted definition of AM is the process of layer-by-layer material deposition from a digitalized model [4]. One distinctive feature of AM is the localized control of material deposition [5], allowing for full customization of parts with minimal dependence on tooling [6]. This capability also facilitates the production of a new generation of materials, such as adaptive and multifunctional materials, including recent developments in 4D printing of biodegradable PLA-based composites with enhanced mechanical and shape memory properties [7,8]. The rapid fabrication cycle inherent in AM makes it an appealing technology for various sectors, including bioengineering, aeronautics, civil engineering, modelling, automotive, engineering, the food industry, and art, among others [9,10,11,12,13]. The widespread adoption of AM in several industries is attributed to the multitude of processes falling within its definition, enabling the printing of a diverse range of materials [14]. For example, Fused Filament Technology or FDM (Fused Deposition Modelling) is a popular and cost-effective method for printing polymeric structures [15,16]. Laser-based technologies, such as SLM (Selective Laser Melting), are employed for processing metallic powders [17]. Stereolithography, another process using a laser source, targets photosensitive resins [18,19]. This technology, characterized by high-resolution processing compared to fused filament, results in highly isotropic structures [20]. Stereolithography operates on the principle of a laser beam scanning the surface of a photosensitive resin in a liquid state [18]. The polymerization of the structure occurs at the spot targeted by the laser beam based on the 2D pattern derived from the slicing step [21]. Although stereolithography achieves high resolution, its lengthy process time is a drawback due to the extensive toolpath scanning [22]. Digital Light Processing (DLP) is another AM method sharing the rationale of stereolithography but addressing the time-consuming scanning process. DLP employs a digital screen projecting an image of the layer under construction, allowing polymerization of an entire layer instead of a single spot [23,24]. Products based on DLP are predominantly used for prototyping, mould fabrication, and consumer products [23,25]. This type of technology can also enable the high-resolution fabrication of mechanical metamaterials with programmable mechanical responses, as demonstrated in recent studies on additively manufactured stair-stepping metamaterials [26].

In this study, we present, for the first time, a successful process of constructing a biosourced blend composed of starch filler and PLA using the photopolymerization route. Most previous studies on PLA–starch composites have focused on thermoplastic processing routes, particularly extrusion. In these approaches, starch generally requires plasticization or thermomechanical processing to ensure compatibility with PLA matrices. For instance, several works have investigated PLA/thermoplastic starch (TPS) blends processed by extrusion, where starch undergoes gelatinization or chemical modification to enable processability and improve interfacial adhesion [27,28,29]. These approaches differ fundamentally from the present work, where native starch granules are directly incorporated into a photosensitive resin without thermoplastic processing. Second, AM of composites through photopolymerization routes such as DLP remains relatively unexplored. Only a limited number of studies have investigated the use of biomass fillers in photosensitive resins for DLP-based composite fabrication. For example, recent studies have incorporated lignin nanoparticles or modified lignin derivatives into photocurable resins, demonstrating improved mechanical performance and printing resolution due to the UV-absorbing and reinforcing properties of lignin fillers [30,31]. Similarly, cellulose-based reinforcements such as microcrystalline cellulose or cellulose nanocrystals have been introduced into DLP resins, showing significant improvements in stiffness and mechanical performance while promoting the development of bio-based photopolymer composites [32]. However, these studies primarily focused on material formulation, printability, or mechanical enhancement, with limited attention given to three-dimensional microstructural characterization of filler dispersion and anisotropy within the printed matrix.

In our study, starch serves as a solid filler in a photosensitive PLA resin, and we discuss the evaluation of composite performance and cost considerations. Photosensitive resins, known for their expensive nature, limit material choices for processing. Blending offers a solution to design structures with specific performance attributes while maintaining reasonable printing costs. Commercial photopolymer resins used in DLP printing typically range between 80 and 150 €·kg^−1^, depending on the formulation and supplier, whereas native maize starch is an inexpensive biomass material with a typical cost below 2 €·kg^−1^. By incorporating, for instance, 10 wt.% starch into the resin formulation, the effective feedstock cost can therefore be reduced by approximately 10%, depending on the base resin price. This estimation is based on the typical market prices of the raw materials and should be considered as an approximate indication rather than a detailed economic assessment. In addition to this economic advantage, starch is a renewable bio-based material derived from agricultural resources, which increases the bio-based fraction of the composite and contributes to improving the environmental footprint of the printed material. Moreover, the use of biomass aligns with the current trend in the plastics industry due to its environmental footprint, high specific mechanical properties, biocompatibility, and transfer properties. For instance, Azmin et al. [33] developed bioplastic films from agricultural wastes with low water adsorption and vapour permeability. Fatima et al. [34] created nontoxic biocomposite materials from bacterial cellulose for biomedical applications, demonstrating their potential in transfer properties while addressing environmental and economic concerns. This study demonstrates that starch filler is a viable choice as it does not compromise the resin’s performance and offers favourable transfer properties. Another motivation for this study is to overcome the challenges of processing starch using other AM routes. Starch is unsuitable for FDM, as heating it beyond the glass transition results in a significant decrease in mechanical stability, rendering it impossible to support any layering during the printing process. Many contributions have addressed various high-added-value applications of PLA/starch composites. For instance, microwave-assisted hydrothermal degradation of starch has been considered to develop PLA/nGO/starch composites [35]. However, to the best of our knowledge, there has been no direct application of PLA/starch utilizing an AM processing route.

## 2. Materials and Methods

### 2.1. Process and Materials

**Figure 1:**
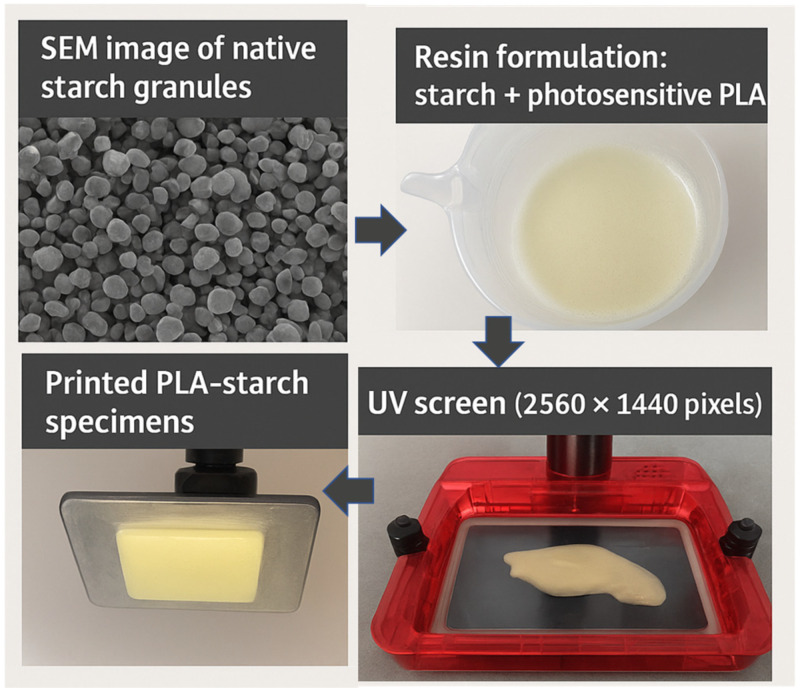
Overview of the printing process using DLP.

The DLP 3D printer (Elegoo Mars (Elegoo France Lespinasse, France) used in this study has a build volume measuring 120 × 68 × 155 mm^3^ (Figure 1). The equipment’s resolution is closely tied to the pixel size of the UV screen, which is about 50 µm. This value represents the nominal resolution of the system and should be considered as an approximate indicator, as the effective resolution may vary depending on processing conditions and material formulation. The resolution achieved is 2560 × 1440 pixels, allowing a building area of 128 mm × 72 mm, and the accuracy in axis positioning is 1.25 µm (Figure 1).

The feedstock materials used in this study comprise a PLA-like photosensitive resin purchased from e-sun 3D France (Lespinasse, France). The detailed chemical formulation of this commercial resin (monomers, oligomers, photoinitiators, and additives) is proprietary and not fully disclosed by the manufacturer. According to the technical documentation provided by the supplier, the material belongs to the family of acrylate-based photocurable resins designed to mimic the mechanical behaviour of PLA after polymerization and is formulated to cure under UV light in the 395–405 nm wavelength range. Table 1 provides the other main characteristics of the PLA photosensitive resin. The filler consists of a native maize starch procured from Unilever (Unilever France—Rueil-Malmaison, France), characterized by a typical granulometry ranging from 10 to 30 µm.

The weight fraction of starch was set at 10%. This filler content was selected to ensure effective reinforcement from the starch granules while limiting the risk of interfacial debonding during processing. This choice is supported by our previous work, where microstructural analysis showed that increasing filler content directly affects photopolymerization through UV light attenuation, leading to porosity development [36]. The starch used in this work corresponds to native dry starch, which was used as received. Because the DLP process operates at room temperature and relies on photopolymerization rather than thermal melting, no gelatinization or thermal degradation of starch occurs during printing. Prior to printing, native starch powder was gradually incorporated at room temperature into the photosensitive PLA resin and mixed under continuous mechanical stirring until a visually homogeneous suspension of starch granules within the resin was obtained. No additional drying treatment was applied to the starch. After mixing, the suspension was used shortly before printing in order to minimize the risk of particle sedimentation in the liquid resin.

Previous investigations on DLP-based composites have identified a threshold filler content beyond which such debonding and related defects are more likely to occur [36]. Parallelepipedic specimens of 80 × 50 × 4 mm^3^ are printed using UV light (the wavelength is 405 nm) with an exposure time of 20 s for the first five layers and 7 s for the remaining ones. The duration of the printing process is 20 min. Regarding cure depth and print resolution, no noticeable deterioration in printing quality or layer definition was observed when incorporating 10 wt.% starch into the photosensitive PLA resin. The printing parameters used in this work (exposure time and layer thickness) remained identical for both the pristine resin and the PLA–starch blend. The successful fabrication of the specimens with the same printing resolution indicates that the presence of starch particles at this loading level does not significantly affect the photopolymerization process or the effective curing depth under the selected processing conditions. All specimens are subjected to a post-curing step consisting of a further 15 min of exposure to UV light after the green state.

**Figure 2:**
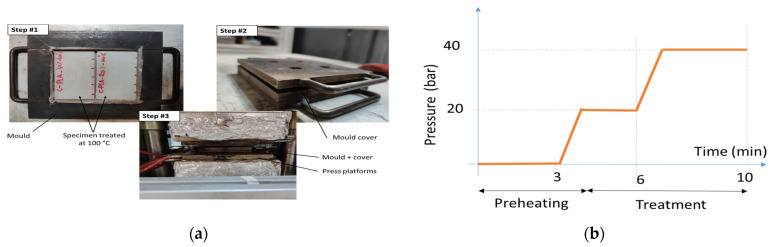
Post-processing of 3D-printed specimens consisting of a thermomoulding step: (**a**) overview of the process; (**b**) applied pressure profile.

The printed specimens are eventually exposed to a post-thermomoulding process at three main temperatures: 50 °C, 100 °C and 150 °C (Figure 2). Figure 2 shows the applied pressure profile at constant temperature and the overview of the post-processing.

The thermomoulding cycle involves two pressure ramps separated by an intermediate plateau, followed by a final holding stage at the maximum pressure. Pressure was applied progressively at a rate of 20 bar/min in two stages. During the first stage, the pressure increases from the initial level to an intermediate plateau of 20 bar over 1 min. The second ramp then raises the pressure from 20 bar to the maximum pressure of 40 bar within the same duration. The pressure is maintained at the intermediate plateau for 2 min, after which it is held at 40 bar for 3 min. The cycle is completed by a cooling stage under pressure prior to demoulding. The total processing time was 10 min. Cooling was carried out in a desiccator at room temperature after demolding, requiring approximately 10 to 15 min to reach thermal equilibrium. Table 2 shows the main processing conditions used in this study. Up to four samples per condition are printed for further analysis. The sample coding system used in Table 2 corresponds to both the material composition and the post-processing temperature. Specifically, the prefix “P” refers to the printed PLA-based material, while “ST” denotes the presence of 10 wt.% starch filler in the formulation. The numerical suffix indicates the thermomoulding temperature applied during post-processing (e.g., 050, 100, or 150 °C), while “000” corresponds to specimens that did not undergo thermomoulding. For example, PST050 designates a PLA–starch blend thermomoulded at 50 °C, whereas P00000 corresponds to pristine PLA printed without starch and without thermomoulding.

### 2.2. Synchrotron X-Ray Microtomography

**Figure 3:**
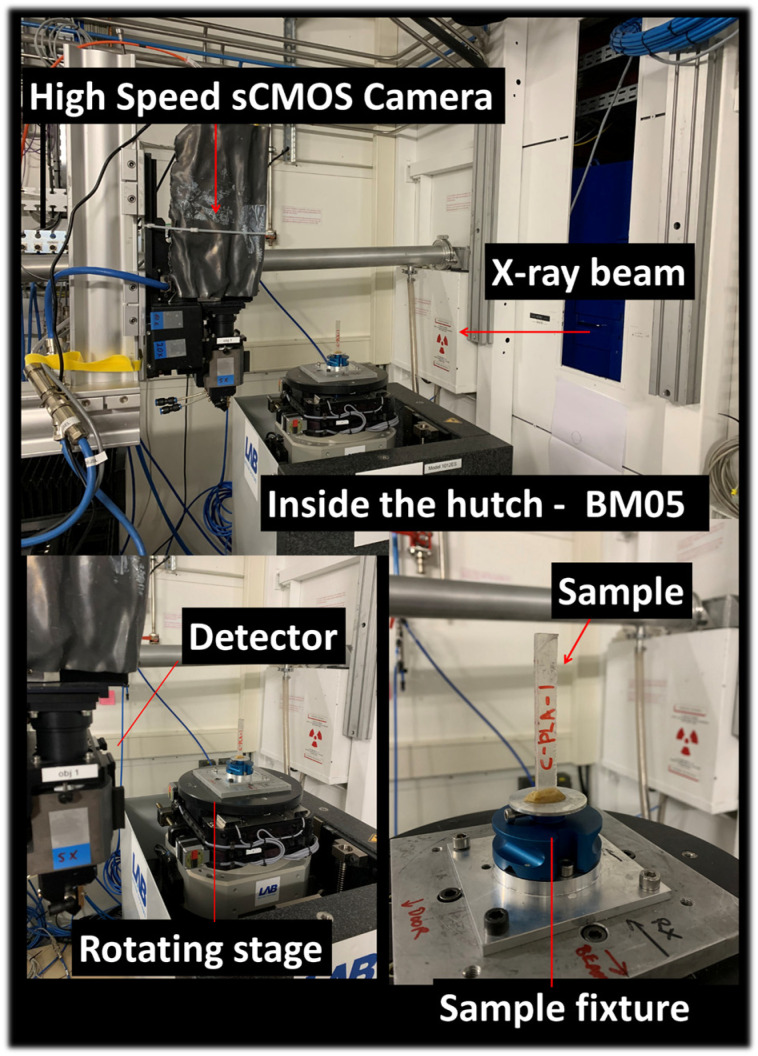
Experimental setup used for synchrotron X-ray microtomography.

Prior mechanical testing, synchrotron X-ray microtomography is used to obtain 3D microstructural information with respect to all formulations. X-ray microtomography provides fully quantitative three-dimensional information, in contrast to conventional imaging techniques such as SEM. The ESRF beamline BM05 in Grenoble, France (Figure 3), is used to acquire the 3D microstructure of all samples using the following parameters: energy of 97 keV, 360° scan range, 1396 mm sample–detector distance, 5000 radiographic images, 101 reference images, 100 dark images, 2048 × 800 pixels detector resolution, 15 ms count time, 15 ms latency time, and a 3.04 µm voxel size. This voxel size corresponds to the resolution limit for defect detection in the present study.

By using a filtered back-projection reconstruction algorithm, we obtain the cross-sections of our sample. In addition, Paganin filtering is used for phase retrieval and to enhance the phase-contrast images. To cover a larger field of view across the sample height, we carry out two successive acquisitions with a small overlap, doubling the acquired volume. The two acquisitions are later stitched with a final volume of 3856 × 3856 × 1600 voxels, covering an acquired volume of 11.72 × 11.72 × 4.86 mm^3^. Image analysis is performed using ImageJ software v. 1.54 from NIH (Bethesda, MD, USA), encompassing image processing tasks such as brightness calibration, conversion to 256 grey-level images, segmentation, 3D rotation, and filtering using opening and closing operators. Additionally, background elimination, phase content determination, anisotropy assessment, and size distribution calculations are part of the image analysis process. In the present study, several quantitative descriptors were extracted from the reconstructed volumes. The starch volume fraction is measured directly, and spatial profiles of starch content are obtained along both the building direction and the in-plane direction, allowing assessment of the material anisotropy. To quantify the size distribution of starch granules dispersed within the PLA matrix, a three-dimensional granulometric analysis was performed on the reconstructed X-ray microtomography volumes. Individual starch particles were identified using a 3D connected-component labelling algorithm. Each connected voxel cluster corresponding to a starch particle is treated as an independent object. From these labelled particles, cumulative distribution of the starch particle volume is derived and related to the printing conditions.

### 2.3. Tensile Testing

Mechanical testing is conducted on both original 3D-printed specimens (PST000) and those undergoing thermomechanical post-processing (PST050–PST150) utilizing two Instron universal testing machines from Instron company (Norwood, MA, USA). The first model is 3366, equipped with a 500 N load cell to study the stiffness of the 3D-printed materials. The second one is a 5566 model, allowing a higher load cell of 1 kN to study the tensile behaviour up to failure. A constant displacement rate of 2 mm/min is employed until reaching the rupture point. Three independent specimens were tested for each processing condition (n = 3). The reported mechanical properties correspond to the mean values ± standard deviation. Statistical comparisons between processing conditions are performed using Student’s *t*-test. It should be noted that the relatively small sample size (n = 3) may limit the statistical power of the analysis and should be considered when interpreting the significance of the results. Even so, this statistical representation allows evaluation of the variability associated with the printing and thermomoulding processes. The tensile tests were performed on rectangular specimens with dimensions of 80 × 10 × 4 mm^3^. A gauge length of 50 mm used for strain measurement corresponded to the central region of the specimen monitored by the digital image correlation (DIC) system, ensuring accurate strain field acquisition during loading.

**Figure 4:**
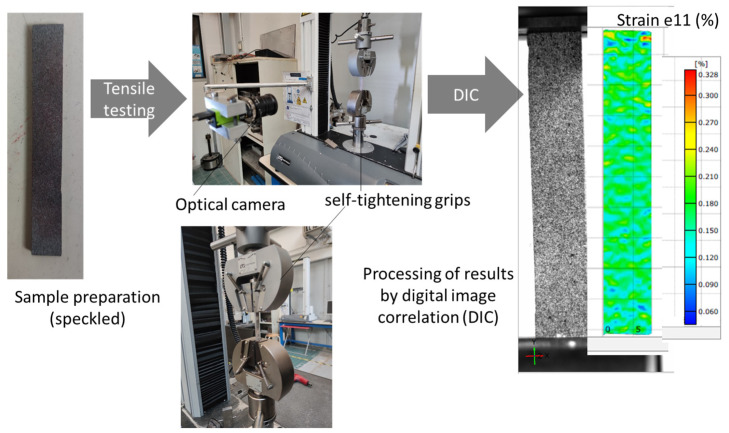
Summary of the tensile system, incorporating the digital image correlation apparatus.

All tested specimens are monitored using a GO-5000M-USB optical camera from JAI company (Copenhagen, Denmark). Before conducting tensile testing, the side facing the camera is coated with a layer of speckle, creating a surface texture with a wide dispersion of spot sizes, including small spots below 100 µm and an average spot size of 200 microns (Figure 4). We relied on speckle quality indicators in the GOM-SNAP software from JAI company (Copenhagen, Denmark). Data processing was carried out using ARAMIS Professional V2.0.1. The speckling process involves a two-step protocol: initially applying a uniform white coating, allowing it to dry, and subsequently applying a layer of black paint on top of the first layer. Surface measurements of displacement fields are obtained from the ongoing optical test recording using the DIC system from GOM France company with a frequency of 3 Hz (Guibeville, France). DIC uses subsets of 19 pixels in size, and a 5-megapixel monochrome output at up to 62 fps, where each pixel corresponds to a physical size of 15 microns. A filter size of 15 pixels is implemented to mitigate noisy strain.

Engineering constants such as Young’s modulus, tensile strength and Poisson’s ratios are determined, and the results are discussed according to the processing conditions. Young’s modulus and Poisson’s ratio are determined within the elongation range of 0.05–0.25% according to the plastics testing norm NF EN ISO 527-1. Determination of the same quantities in a larger range, typically between 0.15 and 0.35%, generates a difference of about 16% and 15% for both quantities.

### 2.4. Thermal Properties Characterization

The thermal characteristics of PLA–starch blends are determined through Differential Scanning Calorimetry (DSC). Thermoanalytical analysis is performed on 30–35 mg specimens using the DSC 4000 from Perkin Elmer^®^ equipment from PerkinElmer U.S. LLC (Shelton, CT, USA). Temperature scanning entails an isotherm stage at 25 °C maintained for 2 min, followed by the first heating stage from 25 °C to 200 °C/350 °C with a heating rate of 10 °C/min, a second holding time of 2 min at 200 °C/350 °C and a cooling stage with the same rate down to 25 °C. The analysis is conducted under a controlled nitrogen atmosphere with a flow rate of 20 mL/min. To assess the thermal history of the studied blends, three heating cycles are employed. The thermal analysis is meant to evaluate both thermal transitions and degradation.

The 3D-printed and thermomoulded specimens undergo analysis using mid-infrared spectroscopy employing a Perkin Elmer spectrometer (Perkin Elmer, Villepinte, France). The spectra are acquired in reflection mode spanning from 450 to 4000 cm^−1^ with a resolution of 4 cm^−1^, with a particular focus on the range 1800 to 800 cm^−1^. Infrared spectra are obtained from 4 additional scans using the Spectrum 10.4.00 software. All spectra within the 1800–800 cm^−1^ range undergo baseline correction, unit vector normalization, and the calculation of mean spectra based on three repetitions using the Spectrum 10.4.00 software. The comparison of these spectra with those of the literature enables the identification of the primary components present in the resin.

## 3. Results and Discussion

### 3.1. Composition of PST Blends

**Figure 5:**
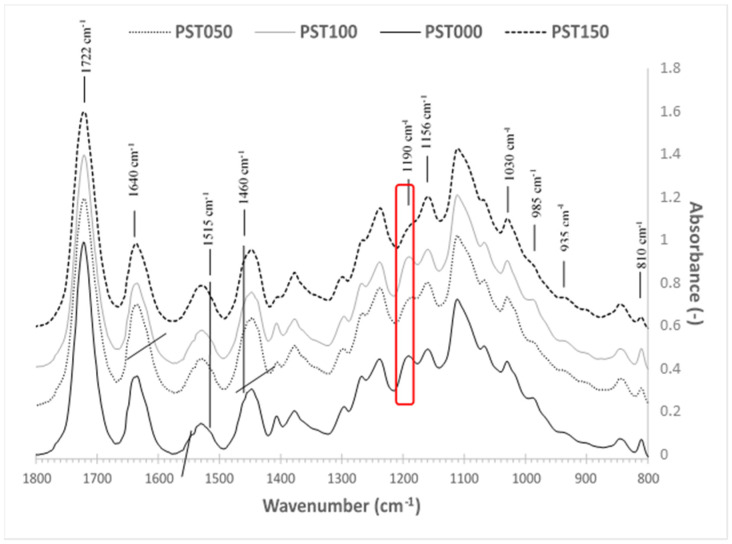
Infrared spectrum of STR blends as a function of the moulding temperature compared to the reference 3D-printed PLA/starch blend with band width assignment.

Figure 5 shows the infrared spectra of the photosensitive PLA blended with starch granules as a function of the moulding temperature. Because the detailed formulation of the commercial resin is proprietary, the interpretation of FTIR spectra relies on the typical chemical signatures reported for acrylate-based photopolymer resins used in DLP printing. The band assignment for all considered blends is summarized in Table 3 based on the literature [27].

Upon analyzing the bending and stretching frequencies, shared components are identified in photosensitive resins [37]. These resins typically consist of a polymeric binder, such as polybutadiene, a polymerizable unsaturated monomer like ethylhexyl acrylate, a polymerization initiator such as benzoin alkyl ether or benzoin, and a thermal polymerization inhibitor like hydroquinone. Additionally, pigments may be included in the composition.

The primary components found in the blended resin consist predominantly of acrylic compounds, including butyl-, ethyl-, and methyl-acrylate. The characteristic infrared assignments for acrylic compounds include the ester C=O band at 1722 cm^−1^, the ethylenic stretching C=C vibration at 1640 cm^−1^, and the CH2 deformation at 1460 cm^−1^. Additionally, peaks associated with in- and out-of-plane C-H bending are observed at 810 cm^−1^. Furthermore, epoxy resin peaks are evident, including the aromatic C=C stretching vibrations bands at approximately 1600 and 1515 cm^−1^, as well as the C-O and C-C stretching bands of the epoxy group at 1035 cm^−1^.

Alongside the peaks associated with epoxy resin, bands characteristic of polysaccharides are identified. These include the C-O-C glycosidic binding at 1160 cm^−1^, the C-O and C-C stretching bands at 1082, 1035, 1027, 1020, and 993 cm^−1^, and the C-CH, C-OH deformation band at 935 cm^−1^. The other remaining peaks are associated with the presence of PLA, such as the carbonyl stretching absorption at about 1750 cm^−1^, and -C-O- in -CH-O- (1182 cm^−1^) and -C-O=O (1127, 1082, 1044 cm^−1^). These assignments are similar to the infrared spectra observed by Wang et al. [27] for thermoplastic dry starch/PLA blends.

A thermal degradation of PLA is observed at 150 °C, as confirmed by the disappearance of the peak at 1190 cm^−1^.

### 3.2. Thermal Behaviour of PST Blends

**Figure 6:**
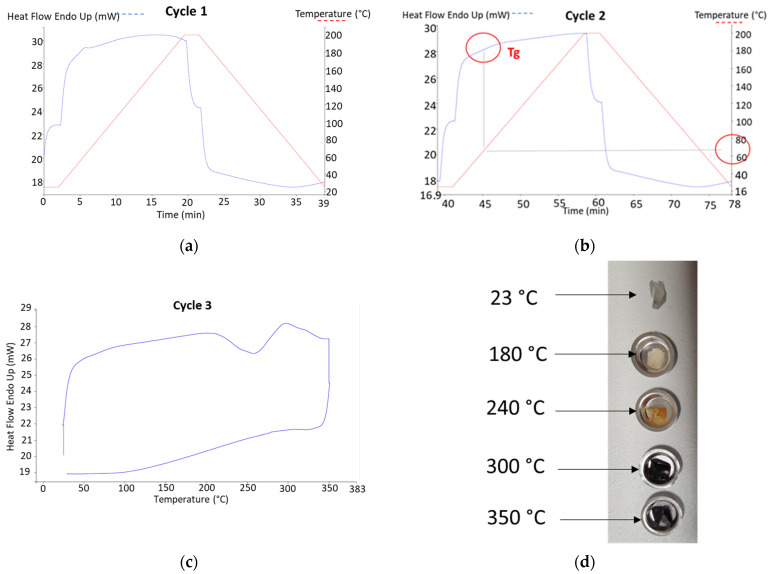
Typical DSC results of photosensitive resin PLA–starch blends: (**a**) first heating cycle used to get rid of thermal history; (**b**) second heating cycle for the determination of glass transition Tg from heat flow curve; (**c**) study of the thermal degradation from the third heating cycle; (**d**) status of the specimen at different heating stages.

Figure 6 shows the DSC results of PST samples for three successive cycles. The primary thermal transitions observed in photosensitive PLA exhibit both endothermal and exothermal effects, with one endothermic transition corresponding to the glass transition of PLA near 70 °C (Figure 6b). We do not observe cold crystallization, generally attributed to PLA close to 110 and 170 °C [28,38,39]. This transition is particularly relevant for the thermomoulding treatment performed in this work, since processing temperatures above Tg can enhance molecular mobility and facilitate structural relaxation in the printed composites.

However, Kaczmarek et al. [38] observed the disappearance of the melting peak close to 153 °C after the first heating cycle. The absence of such a peak can be attributed to a low crystallization ratio, as also observed by Akrami et al. [28]. In addition, Wang and Mano [40] observed that the endothermic peak corresponding to PLA melting does not appear at heating rates below 40 °C/min. In addition, PLA exhibits a degradation behaviour above 300 °C, as also observed by Yoksan et al. [41] as well as Nasseri et al. [39] (Figure 6c). Subsequently, there is a stage involving softening and partial melting of the resin, accompanied by thermal instabilities associated with volatiles and decomposition stages occurring at temperatures exceeding an onset of 210 °C. Palai et al. [29] showed that the PLA in thermoplasticized starch blend exhibited a glass transition temperature of 67 °C, a melting temperature of 149 °C, and a cold crystallization temperature of 114 °C.

Blends formulated with native starch granules introduce additional thermal transitions associated with the organic behaviour of starch, as depicted in Figure 6c, which includes events related to water release and organic decomposition. These occurrences coincide with the thermal transitions of PLA and result in the first and second peaks shifting toward higher temperatures by a minimum of 10 °C, as illustrated in Table 2. The third peak remains consistent, indicating the completion of starch decomposition at temperatures exceeding 330 °C (Figure 6c,d). Wu et al. [42] showed that thermograms of PLA/starch exhibited a glass transition that was not affected by the presence of starch granules. On the contrary, Palai et al. [29] observed that the addition of starch granules in PLA resulted in the decrease in both glass transition and cold crystallization temperatures.

### 3.3. Mechanical Results

**Figure 7:**
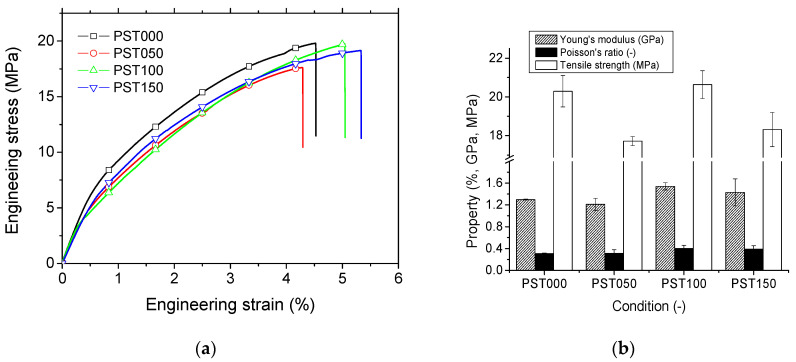
Tensile testing results showing (**a**) overall engineering stress versus engineering strain curves and (**b**) engineering constants for all tested specimens.

Figure 7 shows the typical tensile behaviour of the 3D-printed PST000 specimen. Figure 7a compares the engineering tensile curves of the four studied conditions. All of them exhibit an elastic–plastic behaviour. Although thermomoulding modifies the tensile response of the material, particularly for the condition at 100 °C, the results show that the treatment mainly leads to a moderate increase in stiffness, while the tensile strength slightly decreases compared with the PST000 condition. Figure 7b shows the variation in all engineering constants as a function of the processing conditions. The values correspond to the mean of three independent measurements, and the associated standard deviations are reported to reflect experimental variability. In the reference condition (PST000), tensile tests revealed a tensile strength of 20.30 ± 0.81 MPa, a Young’s modulus of 1.30 ± 0.01 GPa, and a Poisson’s ratio of 0.30 ± 0.01. Post-treatment at temperatures above 50 °C appears to negatively affect the average tensile strength of the specimens, showing a significant decrease of 13% (*p*-value = 0.004). At 100 °C, the tensile strength remains essentially unchanged, while at 150 °C it decreases by 10%; however, this reduction is not statistically significant (*p*-value = 0.09). In contrast, Young’s modulus shows a clear improvement with increasing moulding temperature. Compared to the reference condition (PST000), the average modulus increases by approximately 18%, reaching a maximum of 1.54 ± 0.07 GPa at 100 °C, which is statistically significant (*p*-value = 0.03), although this should be interpreted with caution due to the limited sample size (n = 3). At 150 °C, the modulus decreases relative to 100 °C but remains roughly 10% higher than the reference condition; however, this improvement is not statistically significant (*p*-value = 0.53). Post-treatment with a temperature above 50 °C seems to have a negative effect on the tensile strength of the specimens.

**Figure 8:**
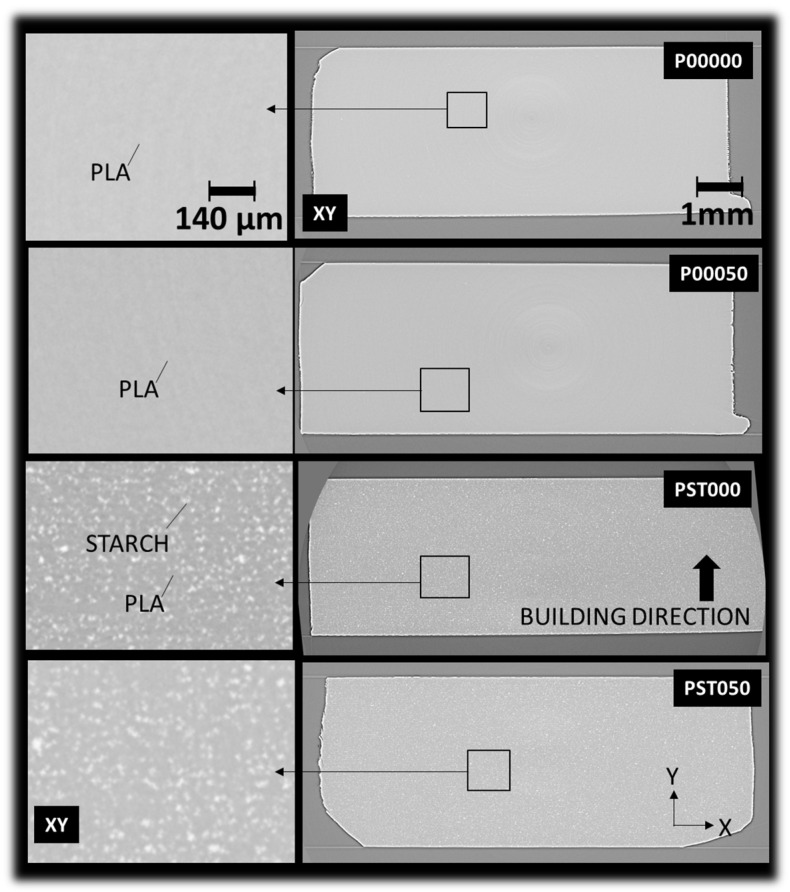
X-ray microtomography images presenting in-plane views that reveal the underlying microstructure of DLP-based materials as a function of processing conditions: pristine PLA (P00000), PLA thermomoulded at 50 °C (P00050), unprocessed PLA–starch blend composite (PST000), and PLA–starch composite thermomoulded at 50 °C (P0ST050).

**Figure 9:**
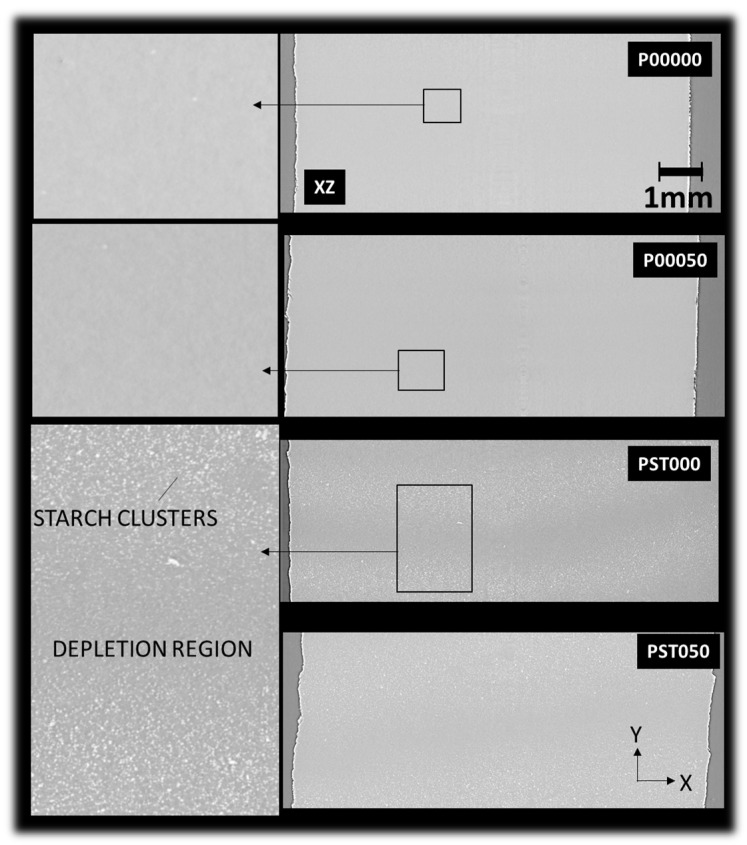
Representative X-ray microtomography images illustrating the phase distribution within the construction plane (XZ).

**Figure 10:**
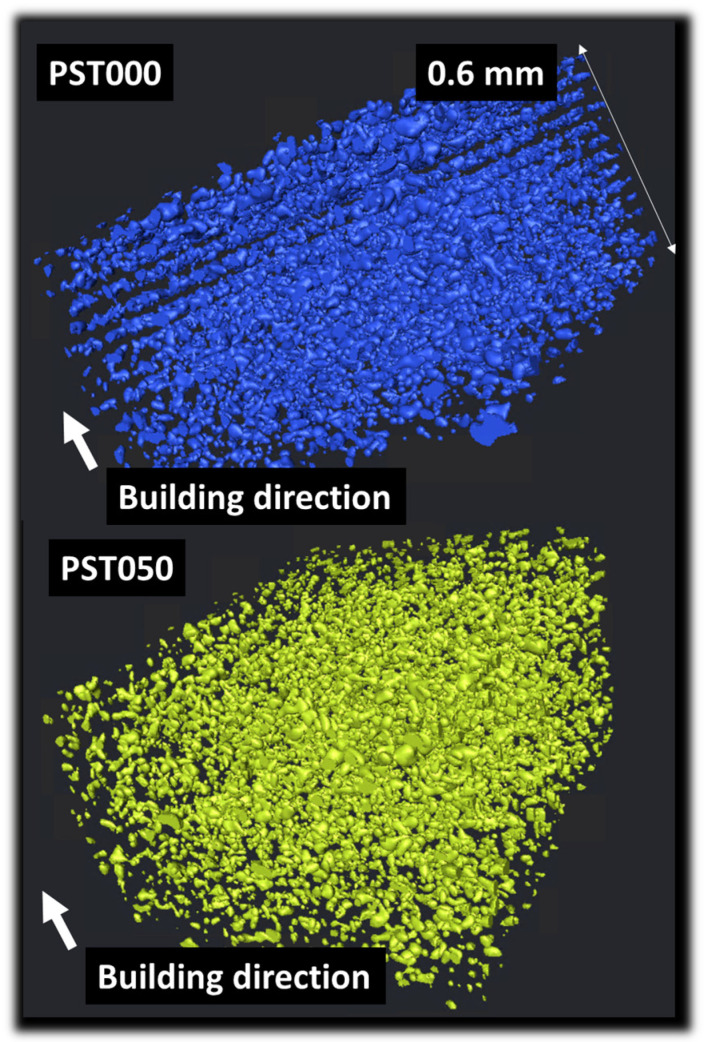
3D visualization of the starch particle distribution within the 3D-printed PLA matrix. Comparison between the untreated (PST000) and thermomoulded (PST050) specimens for a representative volume of 0.94 mm × 0.60 mm × 1.22 mm.

This behaviour can be further explained by the microstructural observations obtained from synchrotron X-ray microtomography (Figure 8, Figure 9 and Figure 10). Thermomoulding at 100 °C, which occurs above the glass transition temperature of PLA (~70 °C), promotes increased chain mobility and facilitates structural rearrangement within the printed material. This results in improved interlayer consolidation and a more homogeneous dispersion of starch particles within the PLA matrix. It should be noted that these interpretations regarding interfacial improvement and particle redistribution are qualitative and inferred from the observed microstructural features obtained by X-ray microtomography. Due to the spatial resolution of the technique (~3 µm), direct measurement of interfacial bonding or nanoscale interactions is not possible. Thus, according to this microstructural observation, such microstructural homogenization is believed to contribute to the observed increase in stiffness. However, this redistribution of the starch phase and the associated relaxation of the initial printed architecture may also introduce localized heterogeneities and stress concentration sites at the microscale. Although no clear interfacial debonding is observed, these microstructural changes can reduce the material’s ability to sustain high stress, thereby explaining the slight decrease in tensile strength. The average increase in Young’s modulus compared to the reference condition (PST000) is about 7%, reaching its maximum of 1.54 ± 0.07 GPa for a moulding temperature of 100 °C. This moderate increase in stiffness contrasts with the slight reduction observed in tensile strength, indicating that thermomoulding primarily influences deformation behaviour and structural consolidation rather than improving the ultimate strength of the material. This behaviour can be explained by the thermomechanical state of the PLA matrix during thermomoulding. Since the glass transition temperature of the photosensitive PLA is around 70 °C, the treatment at 100 °C occurs in the rubbery region of the polymer. In this regime, increased chain mobility promotes stress relaxation, interlayer consolidation of the DLP-printed structure, and improved interfacial contact between the PLA matrix and starch granules [43]. These mechanisms facilitate microstructural rearrangement and contribute to the observed increase in stiffness. In contrast, at 50 °C, the temperature remains below Tg, limiting polymer chain mobility and reducing the efficiency of thermomoulding in improving the mechanical response.

A similar trend is observed for Poisson’s ratio, for which the average improvement with respect to the reference condition is 18% and its maximum value of 0.40 ± 0.06 is achieved at 100 °C. However, this evolution remains statistically non-significant (*p*-value = 0.18), and the same observation holds for PST050 (*p*-value = 0.95) and PST150 (*p*-value = 0.22). The trend of variation in the tensile strength is not obvious to capture. The irregular fluctuations indicate a reduction of 7% with respect to the reference condition (20 ± 0.8 MPa).

### 3.4. Microstructural Interpretation

The X-ray microtomography images in Figure 8 present in-plane (XY) views of DLP-fabricated samples under different printing conditions. This analysis aims to understand the effect of processing conditions and composition on their internal microstructure.

In these views, the *Y*-axis corresponds to the building direction. The pristine PLA sample (P00000) exhibits a homogeneous and compact morphology, with no evidence of defects or phase separation. Likewise, PLA thermomoulded at 50 °C (P00050) retains a uniform structure, suggesting that mild thermal post-processing does not notably affect the material’s microstructural integrity. Synchrotron X-ray microtomography analysis also indicates that no detectable porosity is present in the printed specimens (Figure 8). It should be noted that the spatial resolution of the tomographic analysis (voxel size ~3.04 µm) defines the minimum detectable feature size, and therefore pores smaller than this scale cannot be resolved. At the resolution of the tomographic analysis, no evidence of micro-cracks, void growth, or interfacial debonding between the PLA matrix and starch particles is observed. The microstructure appears relatively dense, with well-dispersed starch domains. However, sub-micrometric heterogeneities and local stress concentration sites, which cannot be resolved at this scale, may still contribute to the observed reduction in tensile strength after thermomoulding. This observation highlights one of the advantages of the DLP process, where printing from a liquid photopolymer resin promotes the formation of nearly fully dense materials. In contrast, the unprocessed PLA–starch composite (PST000) displays a distinct morphology characterized by dispersed starch-rich domains within the PLA matrix. These domains form a linear pattern, with clusters oriented perpendicular to the *Y*-axis, aligning with the building direction. No signs of interfacial debonding or defects are observed between the two phases. This observation is consistent with the selected filler content (10 wt.%), which represents a compromise between dispersion and process-induced defects, as higher filler contents were found to promote air entrapment and porosity development. The spatial distribution of starch particles observed confirms that no significant sedimentation occurred prior to printing. After thermomoulding at 50 °C (PST050), the starch phase becomes more evenly distributed, indicating improved phase dispersion and a partial loss of the initial directional arrangement along the building axis. This improved phase homogeneity is consistent with the mechanical response observed at higher thermomoulding temperatures, where enhanced stiffness is accompanied by a slight reduction in tensile strength due to microstructural rearrangement.

The X-ray microtomography images in Figure 9 show cross-sectional (XZ) views of DLP-fabricated materials, providing insight into the phase distribution and structural uniformity within the plane of construction. The pristine PLA sample (P00000) displays the same homogeneous and dense internal structure without visible defects or heterogeneities, suggesting a free-of-defect structure in the three main directions.

Similarly, PLA thermomoulded at 50 °C (P00050) maintains a comparable microstructure, confirming that mild thermal treatment does not significantly modify the morphology of the printed PLA in the building plane. In contrast, the unprocessed PLA–starch composite (PST000) reveals a clear two-zone morphology: a central region rich in starch clusters and adjacent depletion zones with reduced starch content near the edges. This phase segregation suggests that the DLP fabrication process induces partial migration or uneven distribution of the starch phase during curing. After thermomoulding at 50 °C (PST050), this heterogeneity is notably reduced, with a more uniform dispersion of the starch phase throughout the matrix. This indicates that thermomoulding promotes phase redistribution and improved structural homogeneity, likely due to enhanced mobility and interdiffusion of the PLA and starch phases during heat treatment.

To further investigate the spatial distribution of starch granules within the printed materials, 3D phase segmentation was carried out. Figure 10 presents 3D renderings illustrating the distribution of starch particles before and after thermomoulding. The snapshots represent a representative volume of 0.7 mm^3^ (i.e., 0.94 mm × 0.60 mm × 1.22 mm) of both PST000 and PST050. The PST000 sample shows a relatively dense and anisotropic structure, with elongated starch clusters aligned normal to the building direction, indicating limited particle connectivity. In contrast, the PST050 sample exhibits a more isotropic morphology characterized by well-dispersed starch particles. This change suggests redistribution of the starch granules induced by the applied thermo-mechanical treatment.

**Figure 11:**
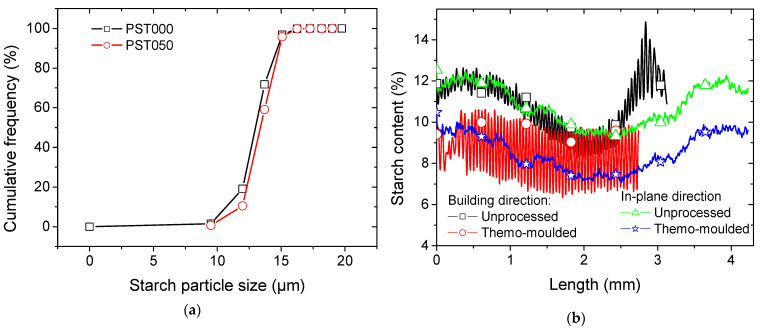
3D image processing results. (**a**) Particle size distribution of starch granules obtained from 3D tomographic analysis. (**b**) Axial distributions of starch filler content along the building and in-plane directions for both unprocessed and thermomoulded DLP-fabricated PLA–starch composites.

To further quantify the morphology of the dispersed starch phase, a 3D granulometric analysis was performed on the segmented tomographic volumes. The particle size distribution obtained from this analysis indicates that most starch particles exhibit equivalent diameters within the micrometric range expected for native maize starch granules (Figure 11a). The cumulative distribution curve shows a progressive increase at small diameters followed by a steep rise in the intermediate size range, indicating that a large fraction of particles is concentrated around a characteristic diameter.

The steep slope of the distribution suggests a relatively homogeneous particle population with limited dispersion toward larger sizes. Only a small fraction of particles exhibits larger equivalent diameters, which may correspond to small agglomerates or clusters formed during the mixing stage prior to printing. The median particle diameter remains close to the typical granulometry of native starch powders, indicating that the DLP printing process does not significantly alter the intrinsic morphology of the starch granules. In particular, detailed quantitative particle size metrics for PST000 samples show that D10 ≈ 10.7 µm, D50 ≈ 13.0 µm, and D90 ≈ 14.7 µm. Similarly, the PST050 sample exhibits D10 ≈ 11.9 µm, D50 ≈ 13.4 µm, and D90 ≈ 14.9 µm. These results indicate that both samples possess relatively narrow particle size distributions, with the majority of particles confined within a limited size range. Notably, PST050 shows a slight shift toward larger particle sizes compared to PST000, consistent with the trends observed in the cumulative distribution curves.

These observations confirm that starch remains mainly dispersed as individual particles within the PLA matrix, consistent with the microstructural features observed in the tomographic renderings. The preservation of the original particle size range suggests that the photopolymerization process and the subsequent thermomoulding treatment do not induce significant fragmentation or coalescence of the starch phase.

Figure 11 shows the axial profiles of the starch content in two principal directions: parallel and perpendicular to the building direction.

The first profile represents the variation in starch concentration along the axis aligned with the material deposition or growth direction, highlighting potential gradients induced by the processing conditions. The second profile illustrates the variation in starch concentration within the building plane, providing insights into the homogeneity of the material and revealing possible differences in starch distribution across the cross-section.

The profiles are obtained for a large representative volume of (8.6 × 2.8 × 4.2 mm^3^). This figure presents a comparison of four datasets over length. The *y*-axis represents the measured starch content within the area of the slice at a specific length position. There are two main distinct trends: one shown in red and black, another in green and blue. The red and black dataset shows substantial variability with alternation of low and high values for starch content. For the untreated sample (PST000), the irregular fluctuations reflect the periodic pattern of starch distribution normal to the building direction, where the particle concentration alternates between high and low values as a result of the layer-by-layer deposition inherent to DLP. A similar phase arrangement has been reported in DLP-fabricated composites incorporating the same type of starch fillers within synthetic matrices, as well as in PLA systems modified with lignin particles [36,44]. The average starch particle content was measured at 10.82 ± 1.29%, which aligns well with the nominal volume fraction targeted in the composite formulation, confirming the representativeness of the acquired volume.

In addition to this irregularity, a wavy trend is observed along the building direction, with peak starch contents reaching up to 14%. These peak values correspond to the bottom and top regions of the specimen, which contain a higher concentration of starch particles, while the central region exhibits a comparatively lower content, around 9%.

After thermomoulding (PST050), the irregular fluctuations become more pronounced, indicating a trend toward dispersing starch particles more uniformly in the building direction. The magnitude of variation reaches approximately 3%, with an average starch content of around 9% at the specimen’s centre. Moreover, the reduction in the wavy pattern suggests a diminished anisotropy in starch particle distribution along the building direction, reflecting the homogenizing effect of the thermomoulding process.

In the in-plane direction, the untreated 3D-printed specimen (PST000) also exhibits a similar wavy pattern, suggesting a tendency for starch particles to form localized clusters, as observed in Figure 9. The magnitude of variation remains relatively small, around 3%. Unlike the profiles along the building direction, no pronounced irregular fluctuation is detected in this orientation, indicating a more uniform lateral distribution of starch particles and a lower degree of structural anisotropy within the printed layers. For the thermomoulded specimen, the starch particle concentration along the in-plane direction follows a similar overall trend, but with a noticeable decrease in the average content and a slight reduction in the amplitude of variation to around 2%.

## 4. Conclusions

We successfully demonstrated the potential of utilizing a PLA/starch mixture through the photopolymerization route to reduce the cost of feedstock materials and enhance the environmental sustainability of technical parts produced through additive manufacturing. DLP technology, in contrast to other methods like fused filament deposition, circumvents the need for starch melting, addressing a significant challenge. We have shown the benefit of DLP to 3D print samples incorporating 10% starch filler. The combination of 3D printing and thermomoulding modifies the mechanical response of the material, leading to moderate improvements in stiffness and deformation behaviour, while the tensile strength remains slightly lower than that of the reference condition. The observed tensile performance is primarily influenced by the moulding temperature, with the optimal behaviour obtained at 100 °C, where the processing temperature exceeds the glass transition temperature of PLA, enabling thermomechanical relaxation and improved structural consolidation. In addition, no discernible alteration in the elastic–plastic behaviour of the 3D-printed PLA/starch was observed. X-ray microtomography revealed distinct microstructural differences between untreated and thermomoulded specimens. The untreated samples showed anisotropic starch particle alignment due to the DLP printing process, while thermomoulding led to a more homogeneous and isotropic distribution. This homogenization indicates improved structural uniformity and potentially enhanced mechanical consistency. In conclusion, the filler contents considered here do not significantly affect the photopolymerization process, irrespective of the moulding temperature. Only minor variations were observed in PLA/starch blends processed above 100 °C compared to the reference condition.

## Figures and Tables

**Table 1:** Main characteristics of the photosensitive PLA resin.

Property	Magnitude	Property	Magnitude
Viscosity	150–250 (at 25 °C, MPa.s)	Flexural strength	46–72 MPa
Wavelength	395–405 nm	Flexural modulus	1–1.4 GPa
Density	1.08–1.13 g/cm^3^	Hardness score	80–82 (shore D)
Tensile strength	46–47 MPa	IZOD impact strength	18–40 J/m

**Table 2:** Overview of the processing conditions of PLA–starch blends.

Condition (-)	Starch Content (%)	Moulding Temperature (°C)
P00000	0	-
P00050	0	50
PST000	10	-
PST050	10	50
PST100	10	100
PST150	10	150

**Table 3:** Main assignment of FTIR spectra for the considered PST samples.

Wavenumber (cm^−1^)	Component	Assignment
3000–3600	Starch	-OH elongation vibration
1750	PLA	Strong carbonyl stretching adsorption
1182	PLA	Stretching of the -C-O- bond of the CH-O- group
1127, 1082, 1044	PLA	Stretching of the -C-O- bond of the -C-O-H group
1156 and 1081	Starch	Stretching of the -C-O- bond of the -O-C=O group
1020	Starch	Elongation of the -C-O- bond of the -C-O-C- group in the anhydroglucid cycle

## Data Availability

The raw data supporting the conclusions of this article will be made available by the authors on request.

## Abbreviations

The following abbreviations are used in this manuscript:

AM	Additive Manufacturing
DLP	Digital Light Processing
DSC	Differential Scanning Calorimetry
FDM	Fused Filament Technology
FTIR	Infrared Spectroscopy
PLA	Polylactic Acid
SLM	Selective Laser Melting
UV	Ultraviolet
